# Gait Optimization Control of Spinal Quadruped Robot Based on Deep Reinforcement Learning

**DOI:** 10.3390/s26082407

**Published:** 2026-04-14

**Authors:** Guozheng Song, Qinglin Ai, Lin Li, Xiaohang Shan, Chao Yang, Jianguo Yang

**Affiliations:** 1 College of Mechanical Engineering, Zhejiang University of Technology, Hangzhou 310023, China; songgz@zjut.edu.cn (G.S.); linli@zjut.edu.cn (L.L.); sxh@zjut.edu.cn (X.S.); 2112102261@zjut.edu.cn (C.Y.); yangjg@zjut.edu.cn (J.Y.); 2 Key Laboratory of Special Purpose Equipment and Advanced Manufacturing Technology, Ministry of Education & Zhejiang Province, Hangzhou 310023, China

**Keywords:** spinal quadruped robot, deep reinforcement learning, TD3–CPG, joint incremental strategy, gait optimization

## Abstract

The spine enhances the flexibility of quadrupeds during locomotion. Inspired by this biological mechanism, this study incorporates an actuated spinal joint into a quadruped robot, enabling more natural motion and posture adjustment. To improve the motion stability of spinal robots in complex environments, a deep reinforcement learning framework that integrates a central pattern generator (CPG) with the twin delayed deterministic policy gradient (TD3) algorithm is proposed to optimize the gait motion of the spinal quadruped robot. First, the structure and parameters of the quadruped robot with a spinal joint are analyzed and a CPG coupling model incorporating spinal motion parameters is designed. Subsequently, a TD3–CPG algorithm framework based on a joint incremental strategy is proposed to optimize the robot’s gait, exploring optimal control strategies for terrain adaptation through spinal motion integration. Finally, experiments are conducted on various obstacle terrains to validate the proposed algorithm. Simulation and experiment results demonstrate the effectiveness of the algorithm in optimizing the gait of the spinal quadruped robot, showing significant improvements in walking stability, speed, and terrain adaptability across different terrains.

## 1. Introduction

As a highly flexible and adaptable robotic system, quadruped robots have attracted widespread attention in the fields of automation, robotics, and intelligent control in recent years. Their movement patterns, inspired by animals in nature, enable superior performance in complex environments compared to bipedal or multi-wheeled robots, particularly in uneven, rugged, or narrow terrains [1,2]. However, the development of quadruped robots faces several challenges, especially in motion stability and terrain adaptability [3]. Although traditional rigid quadruped robots exhibit high structural stability, they often suffer from unstable posture and uncoordinated gait in complex environments, which seriously restricts their practical applications [4].

To address these limitations, researchers have recently explored the integration of spine structures into quadruped robots. By incorporating spinal joints, the flexibility and degrees of freedom (DoFs) of the robot are significantly enhanced, improving its adaptability to complex terrains [5,6]. From a dynamic modeling perspective, the introduction of an active spinal joint introduces additional underactuated dynamics, increases the dimensionality of the system’s state space, and creates strong nonlinear dynamic coupling between the trunk and limb segments. This coupling effect means that the motion of the spine directly alters the kinematic workspace of the foot end and the dynamic distribution of the robot’s center of mass (CoM), which in turn affects the force interaction between the limbs and the ground, greatly increasing the complexity of the robot’s dynamic modeling and motion control [7,8,9].

The spinal design enables the robot to adjust its posture and gait more naturally, enabling better navigation of slope changes and terrain undulations. For example, MIT’s Cheetah robot has enhanced mobility through spinal joints, improving flexibility in different gaits [10]. In terms of spinal morphology selection, existing legged robots adopt various spinal configurations, including rigid, single-DoF (pitch/yaw), and multi-DoF flexible spines. A recent comparative study systematically analyzed the impact of different spinal configurations on gait efficiency, dynamic stability, and control complexity, demonstrating that a single-DoF pitch-actuated spinal joint achieves an optimal balance between motion performance enhancement and control complexity, avoiding the dimensional disaster caused by excessive DoFs, while fully exploiting the flexibility of the spine to improve terrain adaptability [11]. Inspired by this, this study adopts an active pitch spinal joint as the core trunk design of the quadruped robot. However, although the spinal design enhances flexibility and increases the robot’s DoFs, its control precision and motion stability still face significant challenges, especially in multi-DoF systems, where control difficulty and coupling significantly increase [12,13].

Traditional quadruped robot control mainly relies on model-based control strategies, which adjust posture and optimize gait by establishing mathematical models [14,15,16]. While these methods enable relatively stable motion control, they have notable limitations [17]. First, the robot models are highly coupled, making the solution process complex. This is especially true for multi-DoF spinal quadruped robots, where the increased model complexity significantly complicates motion control [18,19]. Additionally, model-based methods require extensive manual parameter tuning to adapt to different environments, which limits their flexibility and adaptability in practical applications [20].

In recent years, the rapid development of deep learning has significantly advanced the application of deep reinforcement learning (DRL) in robotics [21,22,23]. DRL enables quadruped robots to learn optimal motion control strategies through trial-and-error interactions with the environment, using deep neural networks (DNNs) to approximate complex policy or value functions in high-dimensional state spaces, without relying on precise physical models, thereby achieving improved adaptability and generalization capabilities [24,25]. Peng et al. [26] from UC Berkeley proposed the DeepMimic method, which uses teaching trajectories and proximal policy optimization (PPO) algorithms to train quadruped robots to perform complex movements such as martial arts and acrobatics. By imitating human or animal movements and combining them with reinforcement learning, this method enables quadruped robots to exhibit human-like flexibility and diversity in dynamic environments. Similarly, Tsounis et al. [27], at ETH Zurich, developed a method that integrates model-based motion planning with DRL, enabling quadruped robots to navigate irregular terrain and maintain balance in challenging environments. To solve the problem of high-impact force generated by the legs of a quadruped robot when landing, Zhang et al. [28], at the Southwest Jiaotong University, developed a fall control strategy based on deep deterministic policy gradient (DDPG), aiming to learn the impedance parameter adjustment strategy of the legs during freefall within a certain height range, and verified the effectiveness of the proposed method through simulation experiments. Furthermore, to accelerate training convergence, Hu et al. [29], from the Beijing University of Chemical Technology, proposed an efficient method that combines DRL with prior knowledge to optimize quadruped robot gait. By leveraging specific gait patterns as prior knowledge and combining it with the distributed proximal policy optimization (DPPO) method, this method achieves faster convergence and higher-speed gaits. The twin delayed deterministic policy gradient (TD3) algorithm incorporates the double Q-learning algorithm into the DDPG algorithm. Zhu et al. [30], from Beijing University of Technology, combined the TD3 algorithm with policy behavior constraints, updated the policy in the direction of the desired behavior, and reduced boundary actions, which improved the learning walking ability of the quadruped robot.

Currently, research on quadruped robot control mainly focuses on basic motion control and gait optimization. In particular, studies on adaptive control using reinforcement learning are still in the early stages [31,32]. While deep reinforcement learning has shown significant promise in simulations, its real-world application still faces several challenges, particularly in terms of sample efficiency, training speed, and environmental adaptability [33,34]. Additionally, most current research concentrates on controlling basic robot gaits such as walking and turning [35]. However, there is limited research on how spine-based quadruped robots can effectively adjust their gait on complex terrains and use their spine flexibility to improve stability and adaptability [36]. Therefore, it is an important issue to optimize the gait control and terrain adaptability of spine-based quadruped robots by combining traditional control methods with deep reinforcement learning.

In response to the limitations in existing research, this paper proposes a deep reinforcement learning framework that combines CPG and twin delayed deterministic policy gradient (TD3) algorithms. This framework aims to optimize the gait control of spine-based quadruped robots and identify the optimal control strategy for adapting to complex terrain. The proposed method generates gait parameters through CPG, designs joint incremental strategy, and utilizes TD3’s dual-network optimization and delayed policy updates to dynamically suppress value overestimation, thereby significantly enhancing robotic stability and terrain adaptation in complex environments. The main contributions of this paper are summarized as follows.

1. A CPG coupling network incorporating spinal joint parameters is constructed based on the structure of a spinal robot to generate robot motion control parameters;
2. A joint increment strategy for different terrains is designed. The deep reinforcement learning network is used to output the trained joint increment signal and the joint angle signal output by the CPG network is superimposed to obtain the joint control signal of the robot;
3. The TD3–CPG algorithm framework is proposed, which constructs the state space and reward function based on the robot’s state and task objectives, and optimizes the robot’s motion performance on complex terrain by increasing control of the spine.

This paper is organized as follows: Section 2 introduces the quadruped robot structure with the added spinal joint, designs a CPG coupling model incorporating spinal joint motion parameters, and analyzes the characteristics of the spinal joint. Section 3 and Section 4 propose the TD3–CPG algorithm framework, based on joint increment strategies, to optimize the gait of the spinal quadruped robot and explore the optimal control strategy for adapting to complex terrain. Section 5 compares the TD3–CPG and DDPG–CPG algorithms by optimizing robot training on different terrains and analyzes their performance. Finally, simulations and experiments are conducted. By comparing the robot’s motion performance using the pure CPG algorithm (without joint angle adjustment), TD3–CPG, and DDPG–CPG algorithms, respectively, the effectiveness of the TD3–CPG algorithm is further verified.

## 2. Materials and Methods

### 2.1. Model Analysis of Spinal Quadruped Robot

**Figure 1:**
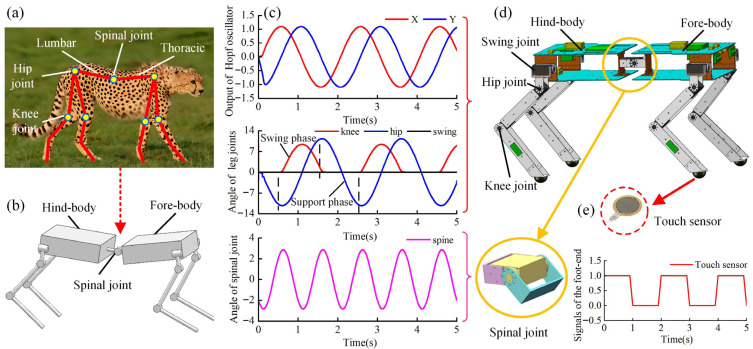
Design of the spinal quadruped robot based on a cheetah: (**a**) cheetah’s body structure; (**b**) simplified model of the spinal quadruped robot; (**c**) the joint curves of the robot; (**d**) structural model of the spinal quadruped robot; and (**e**) touch sensor signals of the robot.

The spinal quadruped robot in this paper is inspired by the body structure and locomotion characteristics of a cheetah, as shown in Figure 1a. Its flexible spine increases the range of motion of the limbs, can increase the speed of movement, and can quickly adjust the body posture during movement through the spine, which helps to maintain body balance and stability. Figure 1b illustrates the robot’s structure, which mimics the cheetah’s body by dividing it into a fore-body, spinal joint, and hind-body, enhancing motion flexibility. Figure 1d shows the structural model: the robot features full-knee leg joints for increased flexibility and maneuverability, an active spinal joint between the fore-body and hind-body, and three degrees of freedom per leg (swing, hip, and knee joints).

This paper designs the control signals of the robot’s leg pitch, hip, and knee joints based on the Hopf oscillator. The control model expression is as follows [37]:

(1) $$\{\begin{array}{l} \dot{x}=\alpha \left(\mu -r^{2}\right)x-\omega y\\ \dot{y}=\alpha \left(\mu -r^{2}\right)y+\omega x\\ r^{2}=x^{2}+y^{2} \end{array}$$

where x and y are state variables; α is used to control the convergence speed of the oscillator; μ determines the amplitude of the oscillator; ω is the frequency of the oscillator; and r is the radius of the state variable.

The legs of the quadruped robot need to move in a specific phase relationship to ensure a stable and effective gait. This phase relationship is achieved through the phase coupling of four Hopf oscillators. The coupling model is given by the following equation:

(2) $$\{\begin{array}{l} \left[\begin{array}{c} \dot{x_{i}}\\ \dot{y_{i}} \end{array}\right]=\left[\begin{array}{cc} \alpha \left(\mu -r_{i}^{2}\right) & -\omega_{i}\\ \omega_{i} & \alpha \left(\mu -r_{i}^{2}\right) \end{array}\right]\left[\begin{array}{c} x_{i}\\ y_{i} \end{array}\right]+\sum_{j=1}^{4}R\left(\theta_{i}^{j}\right)\left[\begin{array}{c} x_{i}\\ y_{i} \end{array}\right]\\ {r_{i}}^{2}={x_{i}}^{2}+{y_{i}}^{2}\\ \omega_{i}=\frac{\omega_{st}}{e^{-ay_{i}+1}}+\frac{\omega_{sw}}{e^{ay_{i}+1}}\\ \omega_{st}=\frac{1-\beta }{\beta }\omega_{sw}\\ R\left(\theta_{i}^{j}\right)=\left[\begin{array}{cc} cos\theta_{i}^{j} & -sin\theta_{i}^{j}\\ sin\theta_{i}^{j} & {cos\theta }_{i}^{j} \end{array}\right]\\ \theta_{i}^{j}=2\pi (\varphi_{i}-\varphi_{j}) \end{array}$$

where *i* = 1, 2, 3, 4, $x_{i}$ and $y_{i}$ are the output signals of the *i*-th oscillator; $R\left(\theta_{i}^{j}\right)$ is the phase coupling matrix; $\theta_{i}^{j}$ is the relative phase difference between oscillator *i* and oscillator *j*; $\omega_{i}$ corresponds to the frequency of different oscillators; and $\varphi_{i}$ is the phase of the *i*-th oscillator.

There is a coupling relationship between the legs and spinal joints during the movement of cheetahs. This paper couples the spinal joint layer oscillator and the left front leg pitch hip joint oscillator of the limb layer to design the spinal joint control signal output. The mathematical model is as follows:

(3) $$\{\begin{array}{l} \left[\begin{array}{c} \dot{x_{0}}\\ \dot{y_{0}} \end{array}\right]=\left[\begin{array}{cc} \alpha_{0}\left(\mu -r_{0}^{2}\right) & -\omega_{0}\\ \omega_{0} & \alpha_{0}\left(\mu -r_{0}^{2}\right) \end{array}\right]\left[\begin{array}{c} x_{0}\\ y_{0} \end{array}\right]\\ +\left[\begin{array}{cc} cos\theta_{0}^{1} & -sin\theta_{0}^{1}\\ sin\theta_{0}^{1} & {cos\theta }_{0}^{1} \end{array}\right]\left[\begin{array}{c} x_{1}\\ y_{1} \end{array}\right]\\ \theta_{s}=A_{s}y_{0}+b \end{array}$$

where $x_{0}$ and $y_{0}$ are the output signals of the spinal joint oscillator; $\theta_{s}$ is the robot spinal joint control signal; $A_{s}$ is the spinal joint motion amplitude; and b represents the bias signal.

**Figure 2:**
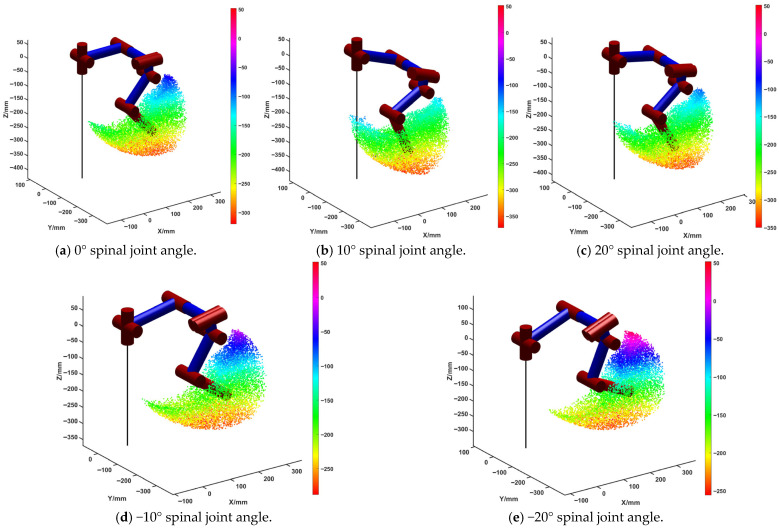
Workspace for different spinal joint angles.

The joint signals of the spinal quadruped robot obtained based on these coupling models are shown in Figure 1c. Using these signals, the robot is able to perform basic movements. In this paper, the robot’s spinal joint is an active pitch joint with the angle range of [−30°, 30°]. Figure 2 shows the robot’s single-leg foot-end workspace at spinal joint angles of 0°, 10°, 20°, −10°, and −20°, respectively.

As shown in the figure, for the forward position, the robot’s foot-end workspace gradually decreases as the spinal joint angle increases from −20° to 20°, with a maximum value of 350 mm when the spinal joint angle is −20°. For the backward position, the robot’s foot-end workspace gradually increases as the spinal joint angle increases, with a minimum value of −120 mm when the spinal joint angle is 20°, representing the furthest backward position. For the highest reachable point, the workspace gradually decreases as the spinal joint angle increases, with a maximum value of 28 mm when the spinal joint angle is −20°. For the lowest reachable point, the workspace gradually increases as the spinal joint angle decreases, with a minimum value of −350 mm when the spinal joint angle is 20°.

Therefore, the joint angles of the spine have a significant impact on the workspace range of the robot’s foot-end. When the spinal joint angles are negative, the robot’s maximum forward reach and highest point increase; when the spinal joint angles are positive, the robot’s maximum backward reach and lowest point increase.

**Figure 3:**
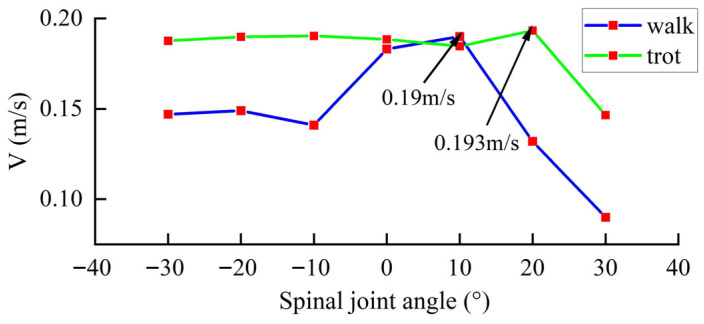
Robot speed for different spinal joint angles.

The spinal angle also has a significant impact on the robot’s movement speed. Figure 3 shows the robot speed corresponding to different spinal joint angles in the robot’s walk and trot gaits. The simulation environment features a flat terrain with a total length of 5 m. The robots have the same step height and a gait period of 1 s. It can be seen that in the walk gait, the robot’s speed increases with the increasing spinal angle, starting from −30°, but reaches a maximum of 0.19 m/s at 10°, after which the speed decreases significantly with further increases in the spinal angle. In the trot gait, the effect of increasing the spinal angle on speed is not significant, remaining stable at around 0.19 m/s, but reaches a maximum of 0.193 m/s at 20°, after which the speed decreases significantly with further increases in the spinal angle.

In summary, by adjusting the angle of the spinal joints, the robot’s workspace and movement speed can be changed. By selecting the appropriate spinal joint angle, the robot can move more stably and quickly. In the subsequent sections, we will further use deep reinforcement learning algorithms to adjust the joint angles of the spinal quadruped robot, thereby enhancing its ability to adapt to different terrains.

### 2.2. Gait Optimization Framework Based on TD3–CPG Algorithm

Although the CPG-based gait planning method can generate stable joint signals and basic motion patterns for spinal quadruped robots, these methods cannot make real-time adjustments based on the robot’s current state, making it difficult to effectively control the robot to complete tasks on different terrains. By incorporating deep reinforcement learning algorithms, the robot can better process sensor data, learn complex strategies through trial and error, make real-time decisions, and dynamically adjust the angles of each joint, thereby improving its adaptability to different terrains.

#### 2.2.1. DDPG Algorithm

**Figure 4:**
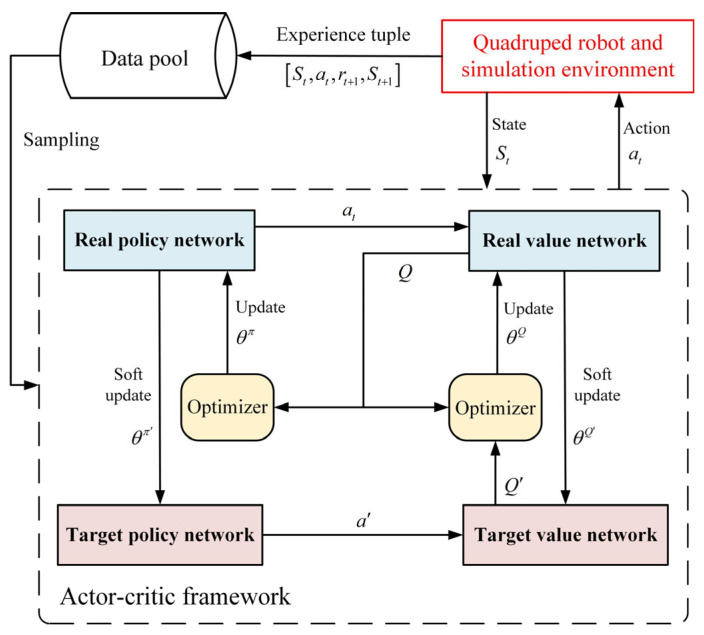
DDPG algorithm flowchart.

DDPG is a classic DRL algorithm for continuous action space tasks, which extends the actor–critic (AC) framework by combining a deterministic policy gradient with deep neural networks [38]. It introduces experience replay and target network mechanisms to improve training stability, making it suitable for solving the complex continuous motion control problem of spinal quadruped robots in this paper. The DDPG algorithm consists of four deep neural networks: two policy networks (real policy network and target policy network) and two value networks (real value network and target value network). The definitions and formulas of each part are shown in Table 1. Figure 4 shows a flowchart of the DDPG algorithm.

Where $s_{t}$ is the given current state; $\theta^{\pi }$ is the real policy network parameter; π represents the real policy network; $a_{t}$ is the action selected by the real policy network; $\theta^{Q}$ is the value network parameter; and Q is the action value function.

The DDPG algorithm introduces an experience replay mechanism that randomly samples training data from a stored pool during training to reduce the correlation between samples. The experience tuple in the data pool is $\left[s_{t},a_{t},r_{t+1},s_{t+1}\right]$, and $r_{t+1}$ is the reward value after the agent performs the action. For the real value network, the target value, which is the correspondence between different states and reward values, is calculated by the Bellman equation:

(4) $$Y=r_{t+1}{+\gamma Q}^{\prime }(s_{t+1},{a_{t}}^{\prime }|\theta^{Q^{\prime }})$$

where γ is the discount factor.

We posit that the Q value output by the real value network is equal to the target value Y, thus we convert it into a supervised learning problem. Regard Y as a label and use the gradient descent method to update the real value network. The loss function is the mean square error between the Q value and the Y value in the current state:

(5) $$L={\left(Q(s_{t},\;a_{t}|\theta^{Q})-Y\right)}^{2}$$

For the action value output by the real policy network, we expect the agent to choose the action value with large value. Therefore, the larger the Q value, the smaller the loss of the policy network. The chain rule is applied to the reward function J to update the real policy network. The reward function and loss gradient of the real policy network are as follows:

(6) $$\{\begin{array}{l} J\left(\theta \right)=Q(s_{t},\;a_{t}|\theta^{Q})\\ \nabla J\left(\theta \right)=\nabla a_{t}\nabla Q(s_{t},\;a_{t}|\theta^{Q}) \end{array}$$

The DDPG algorithm employs a soft update strategy to gradually adjust the parameters of the target value network and the target policy network. This strategy slowly integrates the parameters of the policy network into the target network:

(7) $$\{\begin{array}{l} \theta^{\pi^{\prime }}=\rho \theta^{\pi }+(1-\rho )\theta^{\pi^{\prime }}\\ \theta^{Q^{\prime }}=\rho \theta^{Q}+(1-\rho )\theta^{Q^{\prime }} \end{array}$$

where ρ is the soft update coefficient, which is usually set to a small value.

#### 2.2.2. TD3 Algorithm

The TD3 algorithm is an improvement of the DDPG algorithm, which targets the overestimation bias and error accumulation caused by function approximation in the AC framework, further enhancing training stability and convergence for continuous motion control tasks [30]. Compared with DDPG, TD3 introduces three core improvements.

(1) The maximization operation in DDPG’s target value calculation leads to the overestimation of action values, which will misdirect policy updates. To solve this problem, TD3 adopts two independent target value networks and uses the minimum output of the two networks to calculate the target value, thus suppressing Q value overestimation:

(8) $$\{\begin{array}{l} {{Q_{1}}^{\prime }(s}_{t+1},{a_{t}}^{\prime }|\theta^{{Q_{1}}^{\prime }})\\ {{Q_{2}}^{\prime }(s}_{t+1},{a_{t}}^{\prime }|\theta^{{Q_{2}}^{\prime }}) \end{array}$$

(9) $$Y=r_{t+1}+\gamma \;\underset{i=1,2}{\min}\;{Q_{i}}^{\prime }(s_{t+1},{a_{t}}^{\prime }|\theta^{{Q_{i}}^{\prime }})$$

where ${Q_{1}}^{\prime }$ and ${Q_{2}}^{\prime }$ are target value network 1 and target value network 2; $\theta^{{Q_{1}}^{\prime }}$ and $\theta^{{Q_{2}}^{\prime }}$ are their corresponding parameters.

(2) Function approximation errors will increase the variance of target value estimation and reduce the accuracy of value evaluation. TD3 introduces truncated normal distribution noise into the target action to smooth value estimation, making the value function update more stable:

(10) $$\{\begin{array}{l} Y_{i}=r_{t+1}{+\gamma Q}^{\prime }(s_{t+1},{a_{t}}^{\prime }+\varepsilon |\theta^{Q^{\prime }})\\ \varepsilon ∼clip\left[N\left(0,\sigma \right),-c,c\right] \end{array}$$

where ε is the truncated noise; N is the normal distribution with mean 0 and variance σ; and c is the truncation factor.

(3) The updates of the policy network and value network are interdependent: inaccurate value estimation leads to incorrect policy updates, and inferior policies further aggravate value function error accumulation, forming a vicious circle. TD3 adopts a delayed update strategy: the policy network and target networks are updated only after the value networks are updated *d* times. This strategy reduces the policy network update frequency, provides a more stable value target for policy optimization, and effectively alleviates error accumulation for smoother training.

The pseudo code of the specific process of the TD3 algorithm is shown in Algorithm 1.
**Algorithm 1.** TD3 algorithm1: Initialization: Real value network and target value network $Q_{1}$, $Q_{2}$, ${Q_{1}}^{\prime }$ and ${Q_{2}}^{\prime }$2: Initialization: Real policy network and target policy network π and $\pi^{\prime }$3: Initialization: Data pool capacity N, total iteration number M4: Initialization: Discount factor γ, truncation factor c, initialization state is set to $s_{0}$5:      **for** e = 1 to M **do**:6:      **for** t = 1 to T **do**:7: Select action $a_{t}=\pi (s_{t}|\theta^{\pi })$ according to the prediction policy network8: Execute action $a_{t}$, obtain reward value $r_{t+1}$ and next state $s_{t+1}$9: Store experience tuple $\left[s_{t},a_{t},r_{t+1},s_{t+1}\right]$ in the data pool10: Sample $\left[s_{t},a_{t},r_{t+1},s_{t+1}\right]$ small batch of samples of size n from the data pool Calculation:11: Action after perturbation ${a_{i}}^{\prime }=Q^{\prime }(s_{t+1},{a_{t}}^{\prime }+\varepsilon |\theta^{Q^{\prime }})$12: Update target $Y_{i}=r_{t+1}{+\gamma Q}^{\prime }(s_{t+1},{a_{t}}^{\prime }+\varepsilon |\theta^{Q^{\prime }})$13: Use MBGD to minimize the loss function and update the real value network: $L=\frac{1}{n}{\sum_{i=1}^{n}(Q(s_{t},\;a_{t}|\theta^{Q})-Y_{i})}^{2}$14:      **if** t mod d **then**:15: Use MBGD to maximize the objective function and update the policy network: $J\left(\theta \right)=\frac{1}{n}\sum_{i=1}^{n}Q(s_{t},\;a_{t}|\theta^{Q})$
16: Soft update target network:17:                $\theta^{\pi^{\prime }}=\rho \theta^{\pi }+(1-\rho )\theta^{\pi^{\prime }}$
18:                $\theta^{Q^{\prime }}=\rho \theta^{Q}+(1-\rho )\theta^{Q^{\prime }}$
19:          **end if**20:          **end for**21: **end for**

#### 2.2.3. TD3–CPG Algorithm Framework

In this paper, the TD3–CPG algorithm framework is proposed, integrating CPG with deep reinforcement learning to effectively combine low-level motion generation and high-level decision-making capabilities for controlling the spinal quadruped robot. This approach enhances the robot’s adaptability, decision-making, and learning capabilities in complex, dynamic environments, making it more intelligent, flexible, and efficient.

**Figure 5:**
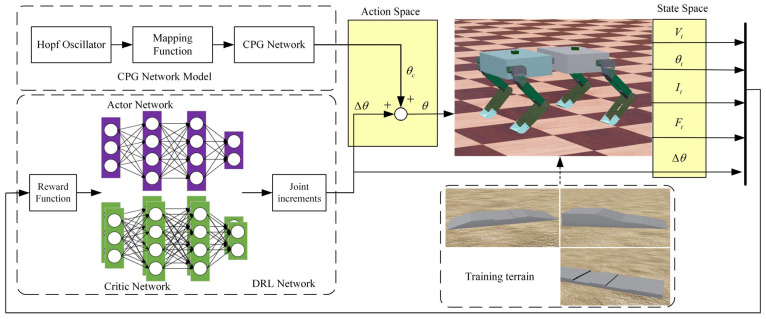
TD3–CPG algorithm framework.

As shown in Figure 5, the TD3–CPG algorithm’s overall framework consists of the CPG network model, deep reinforcement learning network, training terrains, and the spinal quadruped robot. The CPG network model serves as the low-level motion generator, producing stable joint signals for robot gait. The deep reinforcement learning network functions as the high-level decision-maker, outputting gait adjustment signals to respond to environmental changes and varying terrains. The final control signal for the spinal quadruped robot is a combination of signals from the low-level motion generator and the high-level decision-making layer.

The algorithm involves training on various terrains, where the deep reinforcement learning network generates actions based on the robot’s current state. The robot executes these actions to complete walking tasks on different terrains, interacting with the environment to obtain new state information. A reward function calculates the reward value based on the robot’s actions, guiding the update of the policy network. This iterative process of action execution and network updates continues until the robot learns the optimal strategy for adapting to different terrains. To achieve synchronization and control frequency of the hybrid framework, the CPG network and the TD3 policy network operate at the same frequency in each gait cycle. That is, in each simulation time step, the TD3 network generates real-time corrections based on the current robot state, outputs joint increment signals, and superimposes them onto the CPG output to form the final joint control signals.

#### 2.2.4. Joint Increment Strategy

In this paper, the spinal quadruped robot can adjust its state in real time by changing the angles of its four leg joints and spinal joint. During each training cycle, the deep reinforcement learning network iteratively learns to obtain the optimal target joint angles for the quadruped robot to cope with environmental changes, and outputs these target values as joint increments in the CPG network. By directly outputting joint increment signals through the deep reinforcement learning network, the robot can more flexibly respond to environmental changes, exhibiting good dynamic performance and robustness in various complex tasks. The final joint control signal is obtained by superimposing the CPG output joint angle and the DRL output joint increment, with the specific mapping relationship as follows:

(11) $$\{\begin{array}{l} \theta_{si}^{\prime }=\theta_{si}+∆\theta_{3i-2}\\ \theta_{hi}^{\prime }=\theta_{hi}+∆\theta_{3i-1}\\ \theta_{ki}^{\prime }=\theta_{ki}+∆\theta_{3i}\\ \theta_{s}^{\prime }=∆\theta_{13} \end{array}$$

where i=1,2,3,4, $∆\theta_{3i-2}$, $∆\theta_{3i-1}$, and $∆\theta_{3i}$, $∆\theta_{13}$ correspond to the strategic adjustment of the lateral hip joint, hip joint, knee joint and spinal joint, respectively; $\theta_{si}^{\prime }$, $\theta_{hi}^{\prime }$, $\theta_{ki}^{\prime }$ are the final adjusted swing joint, hip joint, and knee joint angle, respectively.

**Figure 6:**
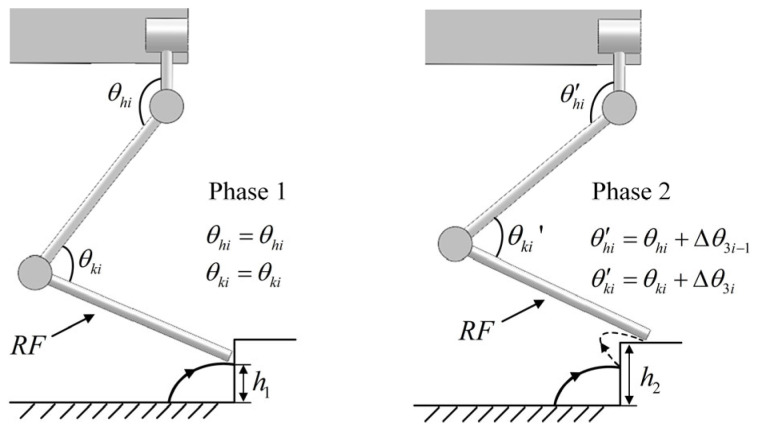
Adjustment strategy for becoming stuck across steps.

As shown in Figure 6, the adjustment strategy for a spinal quadruped robot becoming stuck while crossing a step is illustrated. In phase 1, when the robot attempts to cross a step of height $h_{2}$ (with the RF hip and knee joint signals being $\theta_{hi}$ and $\theta_{ki}$), it becomes stuck after raising the leg only to height $h_{1}$. At this point, the trained DRL network utilizes sensor information to make adjustments and output joint increments, enabling the robot to modify leg joint angles in the next phase and coordinate with the spine joint to increase the leg lift height to successfully cross the step (with the RF hip and knee joint signals being $\theta_{hi}^{\prime }$ and $\theta_{ki}^{\prime }$, and the joint increments being $∆\theta_{3i}$ and $∆\theta_{3i-1}$).

**Figure 7:**
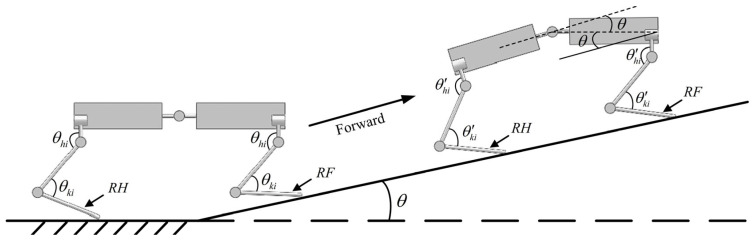
Adjustment strategy for the spinal quadruped robot when climbing.

Figure 7 shows the adjustment strategy for a spinal quadruped robot climbing a slope, involving the right front leg (RF), right hind leg (RH), and the spinal joint. The robot must navigate a slope with an angle of θ. Initially, the robot does not require additional adjustments; however, as the robot gradually climbs the slope, the body gradually tilts, which increases the risk of the robot overturning. The deep reinforcement learning network senses terrain and posture changes by integrating joint position data, posture measurement unit information, and foot force sensor data. On this basis, through the joint increment strategy, the leg joint angle is changed, and the spine joint angle is adjusted to adjust the robot’s posture and lower the center of gravity. Ultimately, the best posture result is obtained in the continuous learning process to ensure that the robot climbs smoothly.

### 2.3. DRL Network for Spinal Quadruped Robot

This section designs the core modules of the DRL network tailored for the gait optimization task of the spinal quadruped robot, including state/action space, reward function with termination conditions, and AC network architecture, to build a task-matched training framework for the proposed TD3–CPG algorithm.

#### 2.3.1. State and Action Space

The state space provides the agent with full observability of the robot’s motion state and environmental interaction information, which is the basis for real-time decision-making. The action space defines the executable control range of the agent, whose design needs to balance task adaptability, learning efficiency and motion safety. To realize accurate gait control and terrain adaptive adjustment, this paper constructs a 42-dimensional state space covering the robot’s motion, interaction and posture information, defined as:

(12) $$s=\left[\theta_{t},∆\theta_{t-1},F_{t},I_{t},V_{t}\right]$$

where $\theta_{t}\in R^{13}$ is the current angle of each joint; $∆\theta_{t-1}\in R^{13}$ is the adjustment from the previous moment; $F_{t}\in R^{4}$ is the foot-end touch sensor signal; $I_{t}\in R^{9}$ is the body posture information, including the posture angle, angular velocity, and acceleration of the posture angle; $V_{t}\in R^{3}$ represents the three-axis velocity of robot’s COM; and $R^{n}$ represents the *n*-dimensional vector.

Among these state components, body posture information and foot-end touch sensor signals are the most critical for controlling the stability of the spinal joint. The body posture information directly reflects the body attitude changes caused by spinal flexion/extension, providing a direct basis for the closed-loop adjustment of the spinal joint; the foot-end touch sensor signal can identify the support phase and swing phase of the legs in real time, ensuring that the spinal joint motion is synchronized with the leg gait cycle, which is the key to avoiding dynamic coupling disturbances between the body and limbs.

The action space is the set of all possible actions the robot can take in interaction with the environment. For quadruped robot gait control, the policy network can output either joint torque or joint angle as the control action. Considering the learning efficiency of the network and the motion control requirements of the spinal joint, this paper takes the joint angle increment as the control output and constructs a 13-dimensional action space (12 dimensions for leg joints, 1 dimension for spinal joint), defined as:

(13) $$\{\begin{array}{l} ∆q_{i}=\left[∆\theta_{3i-2},∆\theta_{3i-1},∆\theta_{3i}\right]\\ A=\left[∆q_{1},∆q_{2},∆q_{3},∆q_{4},∆\theta_{13}\right] \end{array}$$

This low-dimensional incremental control mode reduces the learning difficulty of the policy network while meeting the full-range motion control requirements of the robot.

#### 2.3.2. Reward Function and Termination Condition

The reward function guides the convergence direction of the policy and the termination condition avoids invalid exploration to improve training efficiency; both are necessary to ensure the effectiveness of DRL training.

Aiming at the core goal of realizing the efficient and stable traversal of complex terrain for the spinal quadruped robot, this paper designed a multi-component weighted reward function that comprehensively considers motion duration, forward speed, posture stability, and joint motion smoothness. The specific definition is:

(14) $$\{\begin{array}{l} R=k_{1}r_{t}+k_{2}r_{v}+k_{3}r_{yaw}+k_{4}r_{body}+k_{5}r_{q}\\ r_{t}=\frac{T_{s}}{T_{f}}\\ r_{v}=e^{{\left(v_{x}-0.3\right)}^{2}}\\ r_{yaw}=e^{{-\omega_{y}}^{2}}\\ r_{body}={\left\|\omega_{r}\right\|}^{2}+{\left\|\omega_{p}\right\|}^{2}\\ r_{q}={\left\|\theta_{t}-\theta_{t-1}\right\|}^{2} \end{array}$$

where $k_{i}=1,2,3,4,5$ is the weight coefficient for each reward component; $r_{t}$ is the positive reward of time; $r_{v}$ is the positive reward for speed; $r_{yaw}$ is the negative reward of yaw; $\omega_{y}$ is the robot’s yaw angular velocity; $r_{body}$ is the negative reward of the unstable body; $\omega_{r}$ and $\omega_{p}$ represent the robot’s roll and pitch angular velocities, respectively; and $r_{q}$ is the negative reward of joint mutation.

For the penalty terms, we conducted grid search experiments in the range of [0.1, 2.0] for $k_{3}$, $k_{4}$, and $k_{5}$, respectively, with a step size of 0.1, and selected the weight values that enable the policy network to converge the most quickly and for the robot to have the best comprehensive locomotion performance. The final determined weight coefficients are: $k_{1}=0.8$, $k_{2}=1.0$, $k_{3}=0.5$, $k_{4}=0.6$, and $k_{5}=0.3$. Ablation experiments showed that this weight configuration can balance the optimization goals of each component. The robot can maintain a stable forward speed, while effectively suppressing body posture fluctuations and joint angle mutations, avoiding the problem of a single indicator dominating the training process, leading to the degradation of other performances. For example, if the weight of the speed reward is too high, the robot will have excessive stride and body overturning; if the weight of the posture penalty is too high, the robot will tend to stay in place and cannot complete the terrain traversal task.

To avoid invalid exploration caused by robot falls or abnormal postures in training, this paper set three termination conditions. The current training episode was terminated immediately if any condition was triggered. The overall termination condition is defined as:

(15) $$\{\begin{array}{l} t_{stop1}=\{\begin{array}{c} 1\;\;\;\;\;\;\;\;\;\;\;\;\;t=T\\ 0\;\;\;\;\;\;\;\;\;\;\;\;\;t\neq T \end{array}\\ t_{stop2}=(z\leq 0.3)\\ t_{stop3}=(roll\geq \frac{\pi }{5})∥(roll\geq \frac{\pi }{5})∥(roll\geq \frac{\pi }{5})\\ t_{stop}=t_{stop1}+t_{stop2}+t_{stop3} \end{array}$$

where *t* is the current simulation time; *T* is the maximum simulation duration of a single episode; and z is the height of the body from ground. The three conditions correspond to the maximum preset duration, robot fall, and abnormal body posture, respectively.

#### 2.3.3. AC Network Framework

**Figure 8:**
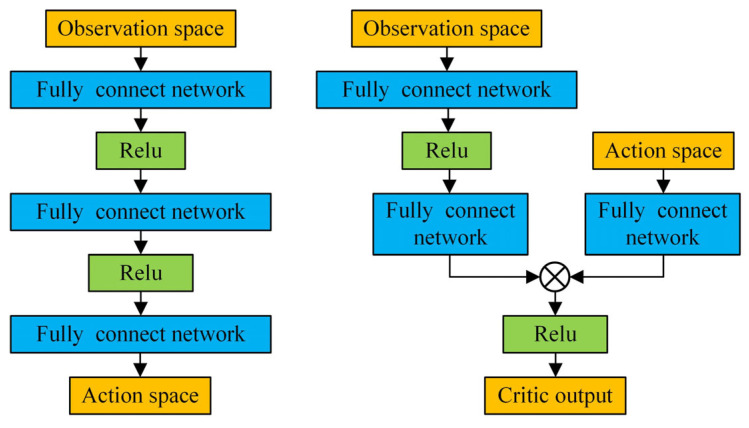
AC frame network structure.

The AC framework used in the TD3 algorithm is shown in Figure 8. The actor network consists of four layers: an input layer, two hidden layers, and an output layer. The input layer represents the state space ***s*** of the spinal quadruped robot, which includes body sensor data and state information. The two hidden layers contain 400 and 300 neurons, respectively, with the ReLU activation function applied to both. The weights and biases are initialized using a uniform distribution, forming a deep, dense neural network architecture capable of processing input features and sensor data. The output layer represents the action space ***A*** of the spinal quadruped robot, which corresponds to the expected angular increments of the robot’s joint motors. The final output values are constrained [−1, 1] using the tanh function.

The two critic networks have the same structure, consisting of an input layer, hidden layers, and an output layer. The two hidden layers contain 400 and 300 neurons, respectively, with ReLU as the activation function. The weights and biases are initialized using a uniform distribution. However, unlike the actor network, the input layer of the critic network receives information from two parts: the state space s and the action space ***A***. The output layer then combines the outputs from hidden layers 1 and 2 for the state space ***s*** and adds the output of hidden layer 2 for the action space ***A***, thus integrating state and action information to more accurately estimate the effectiveness of the policy network.

## 3. Results

In order to evaluate the effectiveness of the TD3–CPG algorithm, we trained a 13-DOF spinal quadruped robot on multi-slope terrain, continuous uneven terrain, and slope uneven terrain. We compared the training results with the DDPG–CPG algorithm and further compared the walking performance in simulations and experiments. Additionally, to demonstrate the role of the spinal joint in the algorithm, we included comparisons using the pure CPG method—specifically, the robot’s walking performance when the spinal joint is at 0° or a fixed angle.

### 3.1. Training and Simulation

**Figure 9:**
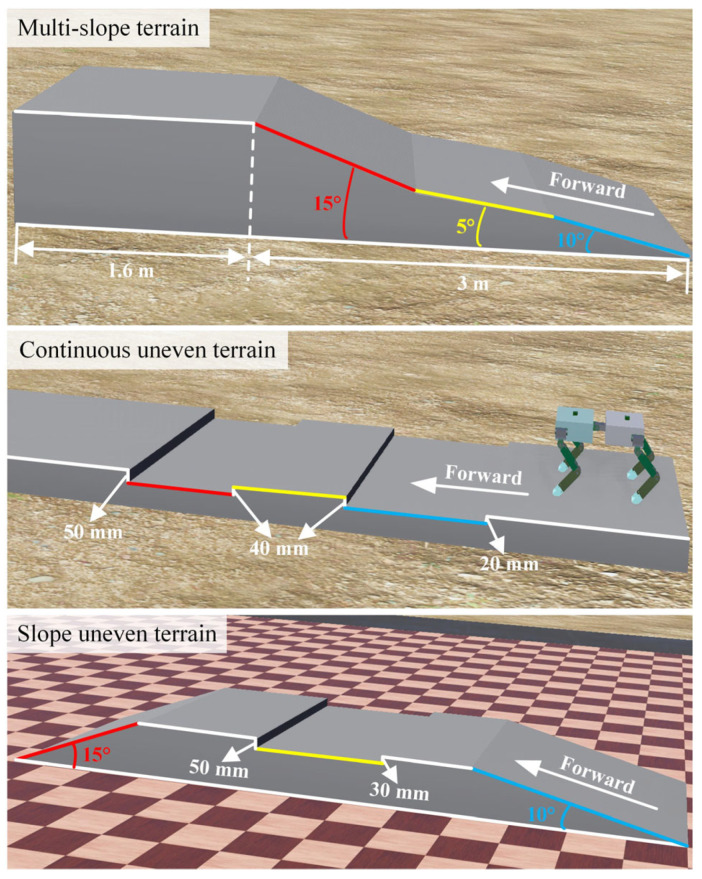
Terrain for training and simulation.

The spinal quadruped robot and terrain environment, built in Webots platform, is shown in Figure 9. The body model of the spinal quadruped robot was established based on its structural characteristics, the size and mass of each joint, the moment of inertia and the spring damping coefficient. The body sensors of the spinal quadruped robot include foot-end force sensors, IMU, and joint encoders. The model parameters are shown in Table 2.

**Figure 10:**
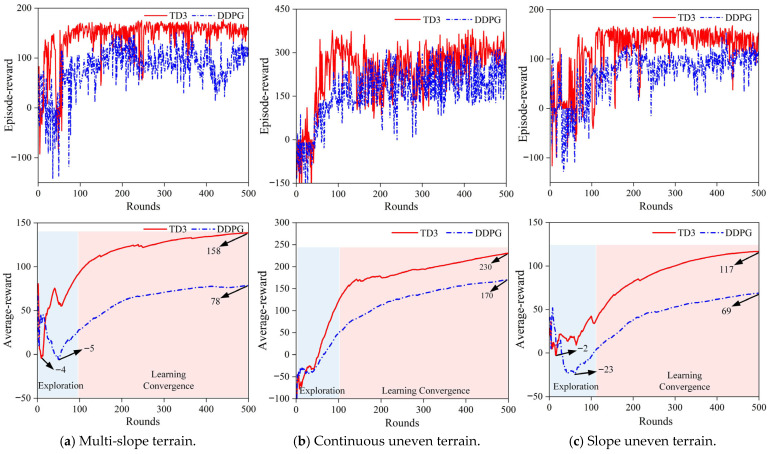
Reward value and average reward value per round on different terrains.

There are three types of terrain for training and simulation in Figure 10. The multi-slope terrain consists of slopes with slopes of 10°, 5°, and 15° upslope; the continuous uneven terrain consists of 20 mm concave, 40 mm convex, 40 mm concave, and 50 mm convex; the slope uneven terrain consists of 10° upslope, 30 mm concave, 50 mm convex, and 15° downslope.

The system control loop frequency is 100 Hz, and the maximum number of steps per round is 600. The quadruped robot was trained for 500 rounds on three different terrains. Figure 10 shows the reward value curve for each round of training across different terrains (the cumulative reward value per round reflects the robot’s learning progress and movement performance). In the early training stages, the performance of both algorithms was similar, with significant fluctuations; the reward value per round was sometimes less than 0. This is because the early policy network will take some bad action values while exploring the action space as much as possible to find the optimal action value, which will cause the robot’s walking effect to be good and bad. However, as the exploration process completes, the policy network gradually determines the update direction and begins to enter the learning convergence stage. During this stage, the robot’s reward value per round significantly improves.

Additionally, because TD3 uses clipped double Q-learning, it effectively avoids the problem of Q-value overestimation, reducing the negative effects of overestimation on policy network updates and subsequent training. As a result, the action values taken are more reasonable and TD3 is less likely to fall into local optima compared to DDPG. In the training, this manifests as avoiding the excessive bias that could lead to an “aggressive” policy such as sudden changes in robot joint angles or excessive strides.

Moreover, due to the double delay update mechanism in TD3, the policy network updates more slowly than the value network, making the policy network training more stable. This further ensures the robot’s stability during movement. As shown in Figure 10, in the later stages of training convergence, the reward value variance is as follows: in multi-slope terrain, the variances for TD3 and DDPG are 20 and 40, respectively; in continuous concave terrain, the variances are 110 and 160, respectively; and in slope concave terrain, the variances are 28 and 65, respectively. This indicates that the TD3 algorithm exhibits lower volatility and is more stable overall.

As shown in Table 3, the convergence reward values of TD3 algorithm in multi-slope terrain, continuous uneven terrain and slope uneven terrain are 160, 314 and 144, respectively, which are significantly higher than the convergence reward values of DDPG algorithm in the three terrains. A higher reward value means that the robot has received better positive feedback during the learning process and has received more rewards. This indicates that the spinal quadruped robot trained with the TD3 algorithm has learned better control strategies, resulting in superior walking performance and stability in all three terrains compared to the DDPG algorithm.

In terms of training convergence speed, the TD3–CPG algorithm reached a stable convergence state after about 500 training episodes in all three terrains. Compared with the recent research on RL-based control [7], the proposed TD3–CPG algorithm with the CPG-based warm start reduced the number of training episodes required, showing a significant advantage in training efficiency. This is because the CPG network provides a stable and biologically plausible baseline gait for the RL framework, avoiding the random exploration of the entire action space in the early training stage; the joint increment strategy limits the learning space of the policy network to the correction of the baseline gait, which greatly reduces the exploration difficulty and accelerates the convergence speed.

Figure 10 shows the average reward value of the two algorithms for different terrain training. It can be seen that in the first 100 rounds, the average reward value of the exploration process of the policy network fluctuated and even dropped to a negative value. The early process of using the TD3–CPG algorithm to train a spinal quadruped robot in a slope uneven terrain showed problems such as stepping on the spot, joint shaking, yaw, and even walking out of the slope and leg stuck. In the 100–500 rounds, which belong to the policy network learning convergence process, the average reward value increased steadily and finally converged to a stable level: in multi-slope terrain, TD3 and DDPG finally reached 158 and 78, respectively; in continuous uneven terrain, TD3 and DDPG finally reached 230 and 170, respectively; in slope uneven terrain, TD3 and DDPG finally reached 117 and 69, respectively. The rise of the average round reward curve indicates that the robot’s control strategy was improved during the learning process and that the TD3–CPG has a higher average reward value, which means that the robot has learned a better strategy.

**Figure 11:**
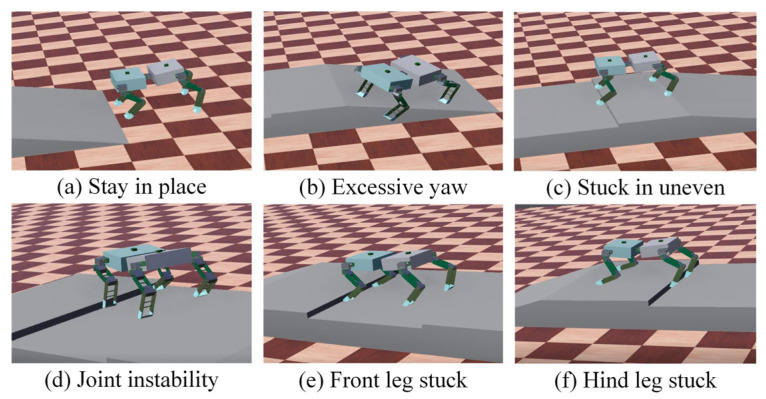
Training process of spinal quadruped robot on slope uneven terrain.

As shown in Figure 11, the training process of using the TD3 algorithm on the slope uneven terrain includes some bad behaviors in the early robot exploration process. In the early stage of training, due to the existence of time rewards, the robot showed the behavior of marking time. In addition, because the strategy needs to explore the action space as much as possible, there will be problems arising from joint jitter and yaw, or even from walking off the slope. As the strategy iteration proceeds, the strategy can gradually adjust the yaw, but it has not yet learned the strategy to deal with the uneven terrain. However, after 500 rounds of iterations, the robot will learn some strategies to successfully pass the slope uneven terrain. Even when the leg is stuck on the convex obstacle, it can immediately adjust the leg joint angle to increase the leg lift height to cross the obstacle.

**Figure 12:**
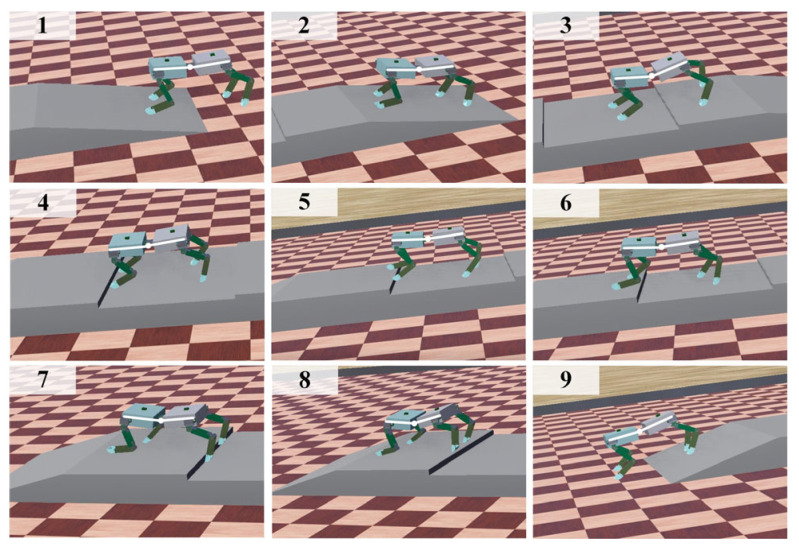
Snapshot of the spinal robot moving on slope uneven terrain.

**Figure 13:**
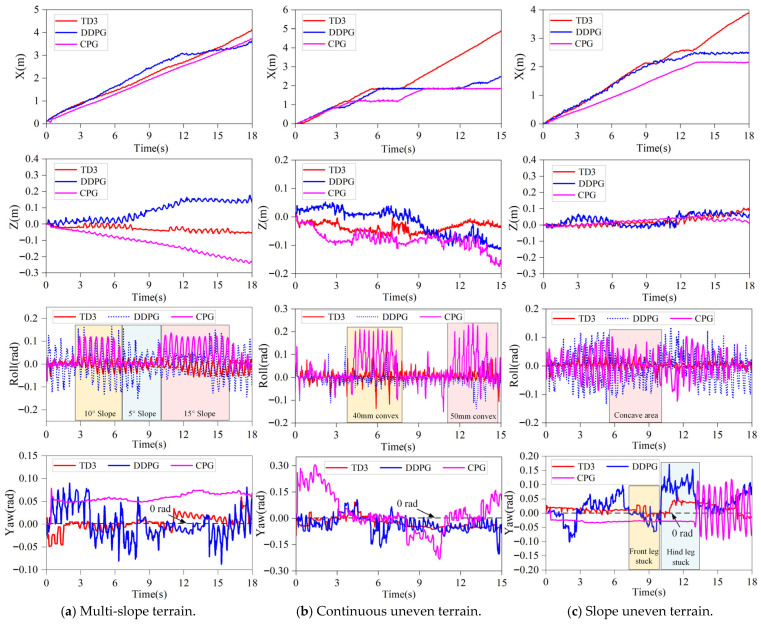
The displacement and attitude angle of the robot moving on different terrains.

In Figure 12, the walking process of the spinal quadruped robot on the slope uneven terrain is shown after 500 rounds of training iterations of the TD3–CPG algorithm. It can be seen that the robot has learned some strategies, enabling it to successfully pass through the slope uneven terrain by adjusting its leg and spinal joints. Furthermore, it can adaptively adjust to avoid obstacles when encountering concave terrain; this process can adjust its yaw angle and the stability of the body. In addition, the robot was further made to walk on multi-slope terrain, continuous uneven terrain and slope uneven terrain using the pure CPG algorithm, the trained TD3 algorithm, and the DDPG algorithm, respectively. All other conditions were kept the same; simulation experiments were carried out to collect data during the robot’s walking process, as shown in Figure 13 and Table 4.

### 3.2. Experiment

**Figure 14:**
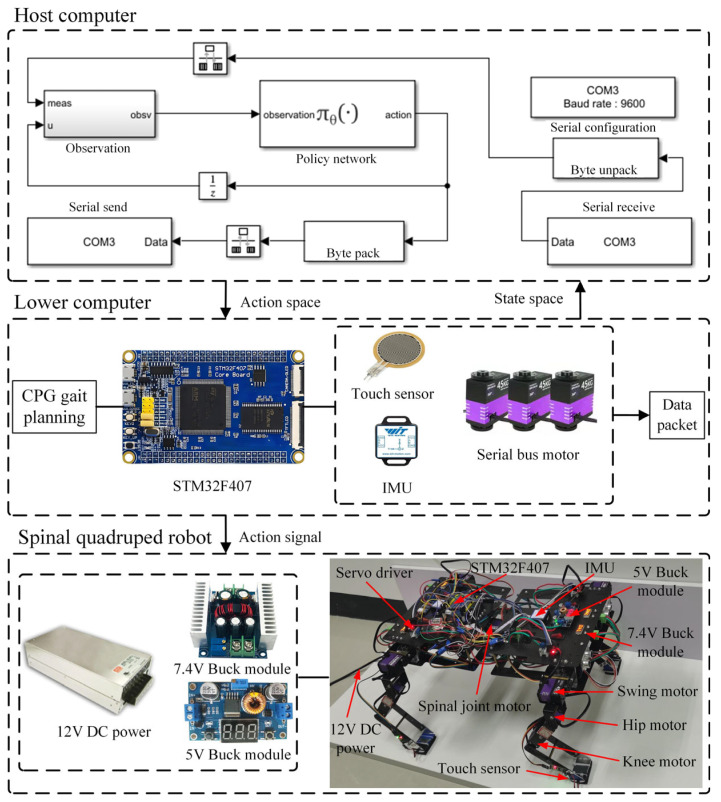
Control system framework of spinal quadruped robot.

Figure 14 presents the control system framework of spinal quadruped robot, which consists of three components: the host computer, the lower computer and the spinal quadruped robot prototype. The host computer implements the robot’s policy network through the following workflow: configuring the serial port (COM3) for host–lower computer communication; receiving data packets containing state space values from the lower computer via the configured serial port; processing these values together with the previous action space values through the policy network; and packaging and sending the newly generated action space values back to the lower computer via the serial port to determine the robot’s subsequent motion strategy.

The lower computer employs an STM32F407 microcontroller as the main control unit. Its functions include: receiving control commands (action space values) from the host computer and combining them with gait planning signals to generate control commands for the serial bus motors at the robot’s joints; controlling the IMU to collect attitude and acceleration data and the touch sensors to acquire ground contact signals; and packaging these data with position feedback from the motor encoders and transmitting them back to the host computer through the serial interface.

The robot’s body is designed in two parts, front and hind, connected by the spinal joint to form an integral mechanism. The connection of the spinal joint has been reinforced. The body is made of carbon fiber material, which has high strength and lightweight characteristics. The 12 V DC power is used to provide the required power supply for the entire robot system. The 5 V buck module adjusts the voltage output by the power supply to the working voltage range of the STM32F407; the 7.4 V buck module adjusts the voltage output by the power supply to the working voltage range of the motor.

Based on simulation results and laboratory conditions, the experimental terrain was built. For the multi-slope terrain, the slope of the first half was 10°, the length is 1 m, and the width was 0.6 m; the slope of the second half was 5°, the length was 1 m, and the width was 0.6 m. The continuous uneven terrain was composed of a continuous 20 mm depression, a 40 mm protrusion, a 40 mm depression and a 50 mm protrusion, with irregular distances between these obstacles. For the slope uneven terrain, the slope of the first half was 10°, the length was 1 m, and the second half was a concave terrain composed of a 30 mm depression and a 50 mm protrusion. The spinal quadruped robot was tested on these terrains using CPG, DDPG–CPG and TD3–CPG algorithms.

**Figure 15:**
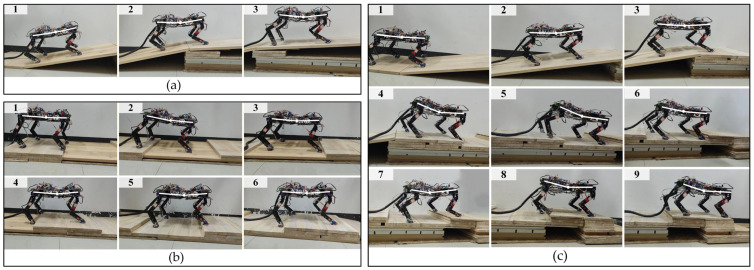
Snapshot of the spinal robot moving on different terrains: (**a**) multi-slope terrain; (**b**) continuous uneven terrain; and (**c**) slope uneven terrain.

**Figure 16:**
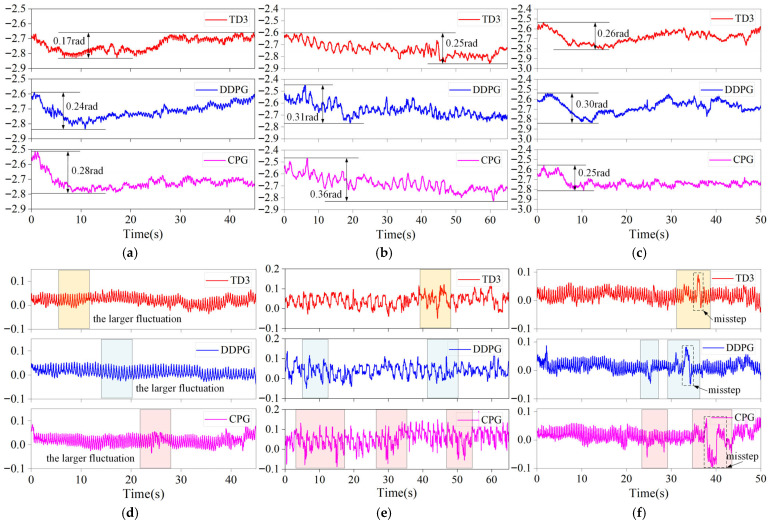
Attitude angle of the robot walking on different terrains: (**a**) yaw angle on multi-slope terrain; (**b**) yaw angle on continuous uneven terrain; (**c**) yaw angle on slope uneven terrain; (**d**) roll angle on multi-slope terrain; (**e**) roll angle on continuous uneven terrain; and (**f**) roll angle on slope uneven terrain.

Figure 15 presents the snapshots of the spinal quadruped robot moving on three different terrains using the TD3–CPG algorithm. Figure 16 shows the yaw and roll attitude angle of the robot in the experiment; the shadows highlight the large fluctuations of the roll angle. Table 5 is a comparison of the robot passing status of the three algorithms on different terrains.

## 4. Discussion

### 4.1. Simulation Discussion

By comparing the horizontal and forward displacements, roll angles, and yaw angles of the robot when the three algorithms are used, respectively, the performance differences of the three algorithms in robot movement were compared.

As shown in Figure 13, in the multi-slope terrain simulation, the spinal quadruped robot can adapt to and pass through the terrain after 500 rounds of training using DDPG and TD3 algorithms. Compared with the robot using the CPG algorithm, those using the deep reinforcement learning algorithms exhibit better adaptability to multi-slope slope terrain, faster travel speed, stronger climbing ability, and a relatively stable body during movement. The robot can recover from disturbances such as adjusting the yaw back to 0 in the case of yaw disturbance. Compared with the two deep reinforcement learning algorithms, the fluctuation range of the DDPG algorithm on low-slope slopes is large; the movement effect of the robot using the DDPG algorithm when passing through higher slope terrains (15° slope) is not good and is even worse than the effect of the CPG algorithm. From the results, the robot using the TD3 algorithm walked a distance of 4.1 m, while the robot using the DDPG algorithm and the CPG algorithm walked a distance of 3.7 m. The TD3 algorithm was the most stable of the three algorithms, passed through the terrain the fastest, and had the smallest final offset distance. When encountering terrain disturbances, the TD3 algorithm demonstrated the smallest attitude angle fluctuations and maintained stable, fast movement, even on steeper slopes. The maximum roll angle fluctuation was reduced by 75% compared to the DDPG algorithm; the final yaw angle was reduced by 80%. These results show that the TD3 algorithm ensures higher stability and faster reactions, providing greater reliability for the robot’s movement.

In the continuous uneven terrain simulation, the TD3 algorithm and DDPG algorithm were used to train the spinal quadruped robot to learn the control strategy for dealing with concave obstacles, achieving better adjustment ability and stability than the CPG algorithm. Compared with the two reinforcement learning algorithms, the performance of the TD3 algorithm is better than that of the DDPG algorithm in adjustment stability and effectiveness. As shown in the figure, during the walking process with the CPG algorithm, the robot’s roll angle fluctuates greatly, with considerable yaw displacement and body yaw angle, preventing it from crossing the 50 mm convex obstacle. The DDPG algorithm performs better, with smaller roll angle fluctuations, yaw displacement, and yaw angle than the CPG algorithm. However, while the robot remains stuck for a long time on the 50 mm convex obstacle, it eventually crosses all obstacles through adjustment. In contrast, the robot using the TD3 algorithm smoothly passes through all concave and convex obstacles, demonstrating superior climbing capability, particularly in overcoming convex obstacles. This shows that during the learning process, the TD3 algorithm better utilized the advantages of the spinal joints. The moving distance of the TD3-trained robot is much larger than that of the other two algorithms, with a very stable passing process and minimal yaw displacement. The roll angle fluctuation of the TD3 algorithm is the same as that of the DDPG algorithm; however, the final yaw angle is reduced by 16.7% compared with the DDPG algorithm, demonstrating the effectiveness and reliability of the TD3 algorithm.

In the slope uneven terrain simulation, compared with robots using the CPG algorithm, robots using the deep reinforcement learning algorithm can more flexibly adjust the stability of the body movement. When facing the yaw problem on the slope terrain, the TD3 algorithm still performs best in the control of the attitude angle; the two reinforcement learning algorithms are basically the same in terms of passing speed. However, when dealing with uneven terrain, the robot using the DDPG algorithm becomes stuck and takes a long time to recover. In contrast, the TD3 algorithm can make quick adjustments, even when the robot’s legs are stuck, allowing it to pass through the entire terrain smoothly and rapidly. Additionally, the TD3 algorithm has relatively small fluctuations in body attitude angle and can quickly adjust the yaw back to zero. Compared with the DDPG algorithm, the roll angle fluctuation is reduced by 65.2% and the final yaw angle is reduced by 85.9%. These demonstrate that the TD3 algorithm is more capable of handling sudden terrain changes.

The above analysis shows that the proposed TD3–CPG algorithm framework effectively solves the “phase-shift” problem required for spinal flexion/extension on different terrains. The CPG network establishes a fixed-phase coupling relationship between the spinal joint oscillator and the leg joint oscillator, which ensures that the spinal motion is inherently synchronized with the leg gait cycle, avoiding the phase mismatch between the body and limbs that is common in RL-based controllers. Meanwhile, the TD3 algorithm outputs joint increments to dynamically adjust the phase and amplitude of the spinal joint motion according to the slope terrain. In addition, the CPG-based underlying gait provides a smooth periodic signal for the joint control, which inherently suppresses the high-frequency oscillations of the RL-based controllers. This is the reason why the TD3–CPG algorithm can maintain excellent stability on slopes and uneven terrains.

### 4.2. Experiment Discussion

In Figure 15a, the spinal robot passes through a 10° slope and then a 5° slope. When the front body and hind body are at different slopes, the robot’s spinal joint is adjusted downward to lower the CoM to increase stability. In Figure 15b, the robot continuously passes through concave and convex obstacles of different heights and distances. When facing concave obstacles, it can adjust the leg joint angles and body posture in time to maintain stability. When stuck by the convex obstacle, the robot can also quickly adjust to cross the obstacle, demonstrating its strong capability in climbing convex obstacles. In Figure 15c, the robot can continuously pass through two different terrains, without any large yaw during the movement process; it can also adaptively change the angle of the spinal joint when switching from the slope terrain to the uneven terrain, so as to better maintain the stability of the body.

In Figure 16, it can be seen that, when passing through multi-slope terrain, all three algorithms will yaw. For the CPG algorithm, due to the lack of adaptation strategy for complex terrain, it cannot effectively cope with terrain changes; therefore, it cannot effectively adjust the yaw angle. The DDPG algorithm showed a large yaw fluctuation during the adjustment process, indicating that the algorithm may be too aggressive or lack adaptability to dynamic environments in some cases. The TD3 algorithm can effectively adjust the yaw angle, making the robot’s yaw angle smaller when going uphill. Its yaw angle fluctuation is reduced by 29.2% compared with the DDPG algorithm; its fluctuation is also the smallest when controlling the robot’s roll angle. According to the data in Table 4, the TD3 algorithm with the best performance reduces the yaw angle fluctuation, final yaw angle error, and roll angle fluctuation by 39.3%, 90%, and 27.8%, respectively, compared with the CPG algorithm. This indicated that the robot using the TD3 algorithm can effectively cope with multi-slope terrains by adjusting the spinal and leg joints and can adjust the yaw angle error while increasing the stability of the robot.

When dealing with continuous uneven terrain, all three algorithms showed obvious yaw. Both the TD3 algorithm and the DDPG algorithm had a tendency to adjust back, while the CPG algorithm kept accumulating yaw. When facing a 50 mm convex obstacle, the TD3 algorithm was able to pass smoothly, the DDPG front legs were able to step over the bump; however, the hind legs would be stuck and the CPG algorithm was completely unable to pass. Compared with the robot using the CPG algorithm, the final yaw angle error of the DDPG and TD3 algorithms was reduced by 21.1% and 93.5%, respectively, and the roll angle fluctuation was reduced by 42.9% and 59%, respectively. Compared with the DDPG algorithm, the TD3 algorithm has a smaller fluctuation in yaw angle; its total yaw amplitude is reduced by 19.4% and the roll fluctuation is reduced by 28.3% compared with the former. This shows that the TD3 algorithm can cope with continuous uneven terrain, can effectively adjust the yaw and roll fluctuation of the body, and can also adaptively adjust the joint angle to cross obstacles when facing high convex obstacles.

When dealing with slope uneven terrain, the TD3 algorithm and the DDPG algorithm can still stably adjust the yaw angle in the slope stage; however, when the terrain suddenly changes to a concave terrain, the performance of the DDPG algorithm deteriorates and the CPG algorithm cannot make effective adjustments during the entire process. It can also be seen from the figure that, when the robot moves to the concave obstacle, it missteps and the body tilts greatly. The TD3 algorithm adjusts the fastest and has the smallest fluctuation. After the robot returns to normal, it passes the 50 mm convex obstacle smoothly. According to the data in Table 5, the best performance is the robot using the TD3 algorithm. Although the roll angle fluctuation is basically the same as the DDPG algorithm, the yaw angle fluctuation and final yaw angle error are reduced by 13.3% and 39.9% compared with the DDPG algorithm. Compared with the CPG algorithm, the roll angle fluctuation and final yaw of the TD3 algorithm are reduced by 24.7% and 45.8%, respectively. This indicates that the TD3 algorithm also has better control effects when dealing with continuously different terrains.

## 5. Conclusions

This paper addresses the problem of optimal gait control for a spinal quadruped robot based on deep reinforcement learning. First, the robot’s gait was planned based on a central pattern generator (CPG) and a multi-layer CPG coupling model was designed to integrate the motion parameters of the spinal joints. A TD3–CPG algorithm framework was proposed to explore the optimal control scheme for the spinal quadruped robot, enabling it to adapt to various terrains by incorporating spinal joint movements. A joint increment strategy was designed to define the action space, including the spinal joint, while the state space and reward function were constructed based on sensor information and task objectives. The spinal quadruped robot was trained on diverse terrains to develop an adaptive motion strategy. The experimental results demonstrated that, compared to the pure CPG algorithm, the TD3–CPG algorithm reduced the robot’s roll angle fluctuation by 27.8% when moving on multi-slope terrain and by 59% on continuous uneven terrain. Similarly, compared to the DDPG–CPG algorithm, the TD3–CPG algorithm achieved reductions of 11.8% and 28.3% on these terrains, respectively. Compared with the other two algorithms, the robot using the TD3–CPG algorithm performed best on slope uneven terrain.

In summary, the TD3–CPG algorithm not only endows the CPG method with real-time adjustment capabilities but also accelerates the robot’s training speed compared to the DDPG algorithm. Compared with other algorithms, the TD3–CPG algorithm can more effectively control the spinal quadruped robot to smoothly cross various terrains, significantly improving its motion stability and environmental adaptability.

In future research, we will consider using parallel reinforcement learning methods to train multiple agents in parallel, make full use of computing resources, shorten training time, and further improve the training efficiency of spinal robots.

## Figures and Tables

**Table 1:** The definitions and formulas of each part of the DDPG algorithm.

Parameters	Formulas
Real policy network	$a_{t}=\pi (s_{t}|\theta^{\pi })$
Real value network	$Q(s_{t},\;a_{t}|\theta^{Q})$
Target policy network	${a_{t}}^{\prime }=\pi^{\prime }(s_{t+1},|\theta^{\pi^{\prime }})$
Target value network	$Q^{\prime }(s_{t+1},{a_{t}}^{\prime }|\theta^{Q^{\prime }})$

**Table 2:** Parameters of the quadruped robot.

Parameters	Value	Unit
Total mass	3.3	kg
Mass of the front body	1.65	kg
Mass of the hind body	1.65	kg
Mass of the femur	0.01	kg
Mass of the tibia	0.01	kg
Length of the femur	116	mm
Length of the tibia	116	mm
Length of the front/hind body	386	mm
Width of the body	302	mm

**Table 3:** Robot training reward value on different terrains.

Stable Reward Value After Convergence	Multi-Slope Terrain	Continuous Uneven Terrain	Slope Uneven Terrain
Convergence reward value of TD3	160	314	144
Convergence reward value of DDPG	118	219	104
Variance of reward value in TD3	20	110	28
Variance of reward value in DDPG	40	160	65

**Table 4:** Comparison of different algorithms on three different terrains in simulation.

Terrain	X-Axis Displacement (m)			Roll Angle Fluctuation (rad)			Final Yaw Angle (rad)		
	CPG	DDPG	TD3	CPG	DDPG	TD3	CPG	DDPG	TD3
Multi-slope terrain	3.7	3.7	4.10	0.12	0.28	0.07	0.06	0.06	0.012
Continuous uneven terrain	1.82	2.47	4.90	0.30	0.15	0.15	0.11	−0.06	−0.05
Slope uneven terrain	2.16	2.49	3.88	0.17	0.23	0.08	0.076	−0.092	−0.014

**Table 5:** Comparison of different algorithms on three different terrains in experiments.

Terrain	Final Yaw Angle (rad)			Roll Angle Fluctuation (rad)			Pass the 50 mm Obstacle		
	CPG	DDPG	TD3	CPG	DDPG	TD3	CPG	DDPG	TD3
Multi-slope terrain	0.223	0.031	0.022	0.072	0.059	0.052	/	/	/
Continuous uneven terrain	0.232	0.183	0.015	0.210	0.120	0.086	No	No	Yes
Slope uneven terrain	0.192	0.173	0.104	0.077	0.057	0.058	No	Yes	Yes

## Data Availability

The original contributions presented in this study are included in the article. Further inquiries can be directed to the corresponding author.
